# The deep-sea pennatulacean genus *Porcupinella* – with the description of a new species from Tasmania (Anthozoa, Octocorallia, Chunellidae)

**DOI:** 10.3897/zookeys.1019.61789

**Published:** 2021-02-22

**Authors:** Gary C. Williams

**Affiliations:** 1 California Academy of Sciences, 55 Music Concourse Drive, San Francisco, CA 94118, USA California Academy of Sciences San Francisco United States of America

**Keywords:** Deep-sea pennatulaceans, key to chunellid species, *Porcupinella tasmanica* sp. nov., sea pens, systematics, worldwide distribution

## Abstract

The recently described deep-sea pennatulacean genus *Porcupinella* was previously known only by the type species, *Porcupinella profunda* from the equatorial eastern Atlantic to the eastern North Atlantic Ocean. New data is provided on morphology, distribution, bathymetry, and related taxa. A second species is added here as well – a new species is described from the Tasman Sea in the southwestern Pacific. The new species, *Porcupinella tasmanica*, is distinguished from *P. profunda* by its distinctive hook-shaped growth form, laterally compressed dorsal keel, and differing regions that are occupied by siphonozooids. A key to the species of the deep-sea pennatulacean family Chunellidae is included based on comparative morphology.

## Introduction

Pennatulaceans or sea pens are a distinctive group of octocoral cnidarians distributed in all oceans of the globe and range in depth from sea level to least 6200 m (Williams 2019). Various aspects of pennatulacean biology regarding taxonomy, morphology and anatomy, ecology, and biogeography have been treated in a variety of publications (Williams 1990, 1992, 1995, 1999, 2011). Of the thirty-seven extant genera of pennatulaceans known, seven of these have been recorded from depths greater than 3000 m (Williams 2019).

The genus *Porcupinella* López-González & Williams, 2011 of the pennatulacean family Chunellidae, was named for the type locality of the first described species – the Porcupine Abyssal Plain, southwest of Ireland in the northern Atlantic Ocean (López-González and Williams 2011). *Porcupinella profunda* López-González & Williams, 2011 was described from twenty-five specimens collected by bottom trawl between 4839 and 4847 m in depth. The genus is differentiated from other pennatulaceans by the dense congestion of paired lateral autozooids on the distal portion of the rachis, and a single terminal polyp between the distal most lateral polyps and the acute distal terminus of the colony.

**Figure 1.**
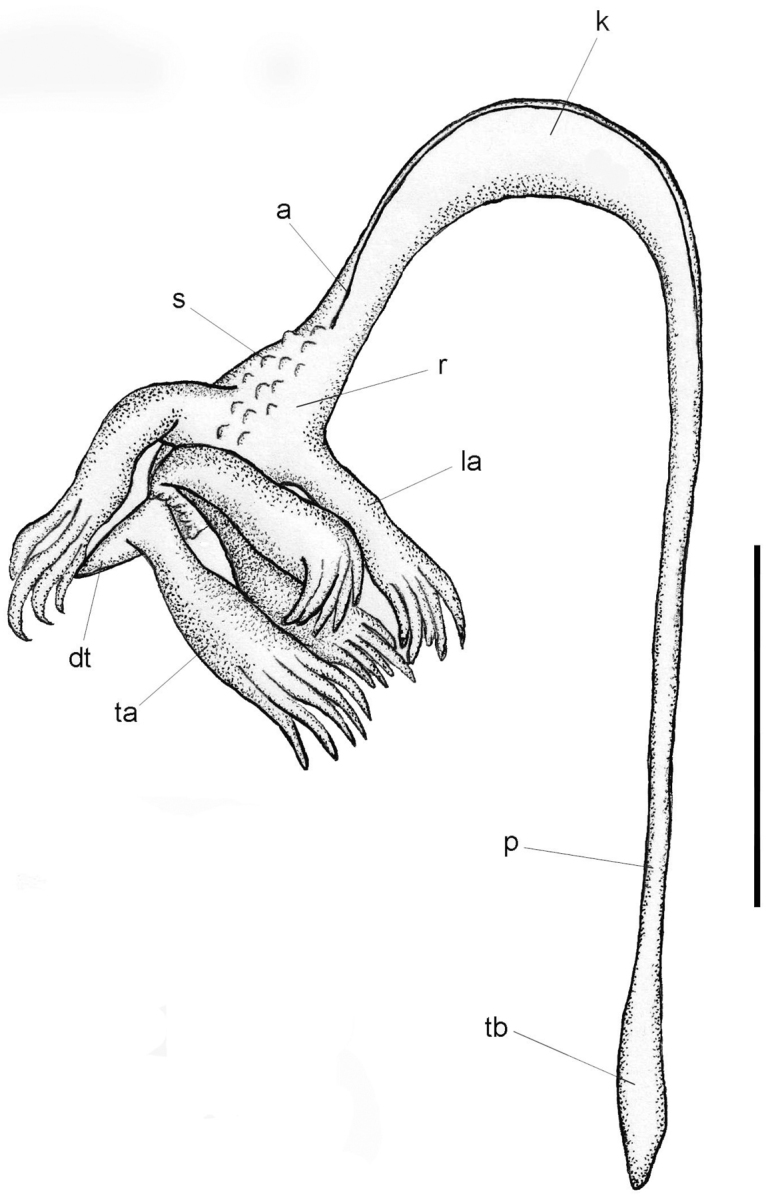
*Porcupinella tasmanica* sp. nov. Diagram of external morphology (Holotype, CASIZ 228434). Abbreviations: a – axis; dt – distal terminus of the colony; k – keel of the stalk; la – lateral autozooid, p – peduncle, r – rachis; s – siphonozooid; ta – terminal autozooid; tb – terminal bulb of the peduncle. Scale bar: 10 mm.

The Chunellidae is a rarely encountered family of deep-sea pennatulacean octocorals (800–5300 m), currently known from the northeastern Atlantic, southwestern Pacific, and western Indian Oceans. The chunellids are morphologically unique in that they possess a terminal polyp, unlike other pennatulaceans. The terminal polyp in chunellids is a single solitary polyp that is morphologically similar to the other paired autozooids in the colony and occupies the most distal position on the polyp-bearing portion of the rachis (Figs 1, 3, 4). Three genera are currently recognized – *Amphiacme* Kükenthal, 1903, *Chunella* Kükenthal, 1902, and *Porcupinella* (Cordeiro et al. 2020a).

An additional species of *Porcupinella*, collected in May of 2017 from the Tasman Sea off the east coast of Australia at a depth of approximately 4100 m, differs from *P. profunda* in several morphological aspects and is described here as a new species.

One noteworthy aspect is that the two species of *Porcupinella* are perhaps the smallest sea pens known, 27–42 mm in recorded length. In addition, the genus *Porcupinella* is known entirely from depths in excess of 4000 m, which is beyond the depth range of most ROV operations, and thus all collected material to date has been acquired by beam or bottom trawl.

The morphological and anatomical aspects of pennatulaceans have been well documented in various publications (Williams 1990, 1995, 1999, 2011). However, the chunellid and umbellulid pennatulaceans do exhibit some significant characteristics that differ other groups of sea pens (Williams 1995).

## Materials and methods

All known material representing the new species was collected by beam trawl between 4114 and 4139 m off the coast of northeast Tasmania in 2017, and is deposited in the invertebrate zoology collection of the California Academy of Sciences. The material was collected by the Australian marine research vessel, the R/V *Investigator*, which is managed by the Commonwealth Scientific and Industrial Research Organization (**CSIRO**) of the Australian government.

Material used in the description of the type species of the genus, *Porcupinella profunda*, was collected by bottom trawl during the BENGAL Program (**BEN**thic biology and Geochemistry of a northeastern **A**tlantic abyssal **L**ocality) in 1997 and 1998 (López-González and Williams 2011: 310).

Illustrations of individual colonies of *Porcupinella* were made using a Nikon 0.7–3.0 zoom dissecting microscope microscope, and were photographed using a Panasonic Lumix DMC-ZS30 camera. Sclerites were isolated from peduncular tissues of the new species using the laboratory technique articulated by Williams and Mattison (2018). The sclerites were observed and photographed using an Olympus CH-2 compound microscope and Panasonic Lumix DMC-ZS30 camera.

Terminology used in this paper conforms to that of Bayer et al. (1983).

## Systematic account

### Phylum: Cnidaria Hatschek, 1888

**Class: Anthozoa Ehrenberg, 1834**

**Subclass: Octocorallia Haeckel, 1866**

**Order: Pennatulacea Verrill, 1865**

**Family: Chunellidae Kükenthal, 1902**

#### Genus: *Porcupinella* López-González & Williams, 2011

##### Porcupinella tasmanica sp. nov.

Taxon classificationAnimaliaPennatulaceaChunellidae

B77C324F-74C0-5BD5-8FFB-89ED551533F7

http://zoobank.org/44193C4B-9A6D-4292-B54A-AA66B72386C0

**Figure 2.**
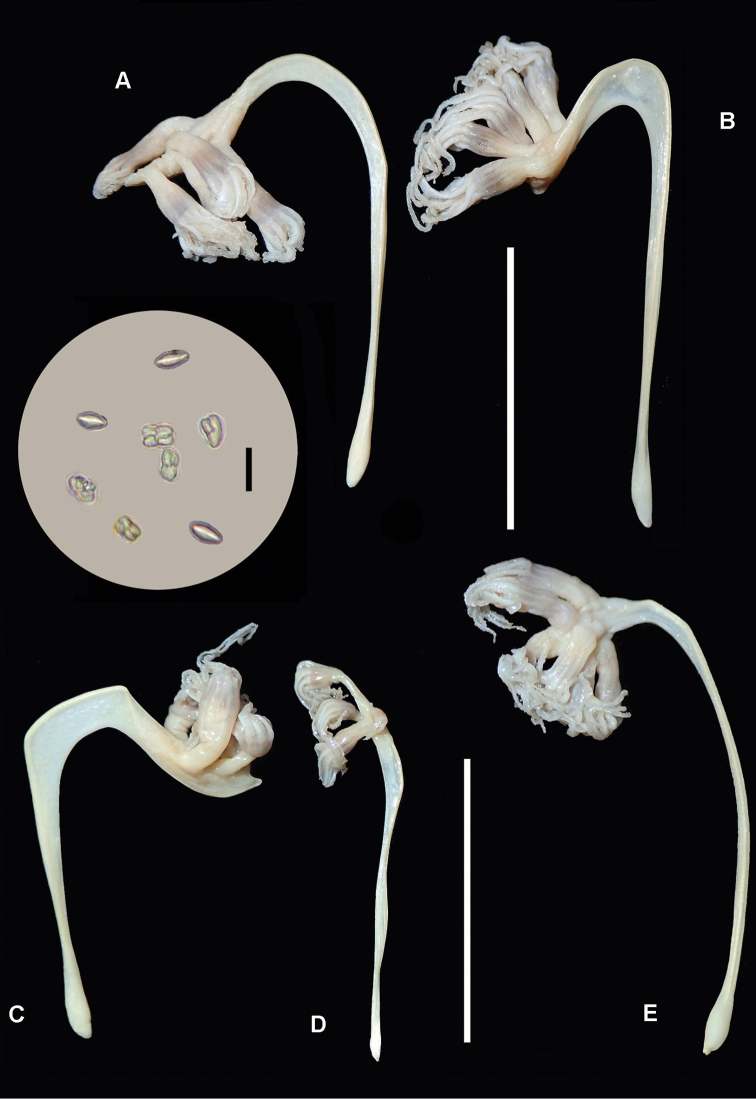
*Porcupinella tasmanica* sp. nov. **A** holotype (CASIZ 228434) **B** paratype (CASIZ 228435) **C–E** non-type voucher specimens (CASIZ 228436); Inset. Composite photomicrographs of minute peduncle sclerites in voucher specimen CASIZ 228436 (Fig. 2E). Scale bars: 20 mm (**A–E**); 0.02 mm (for inset).

**Figure 3.**
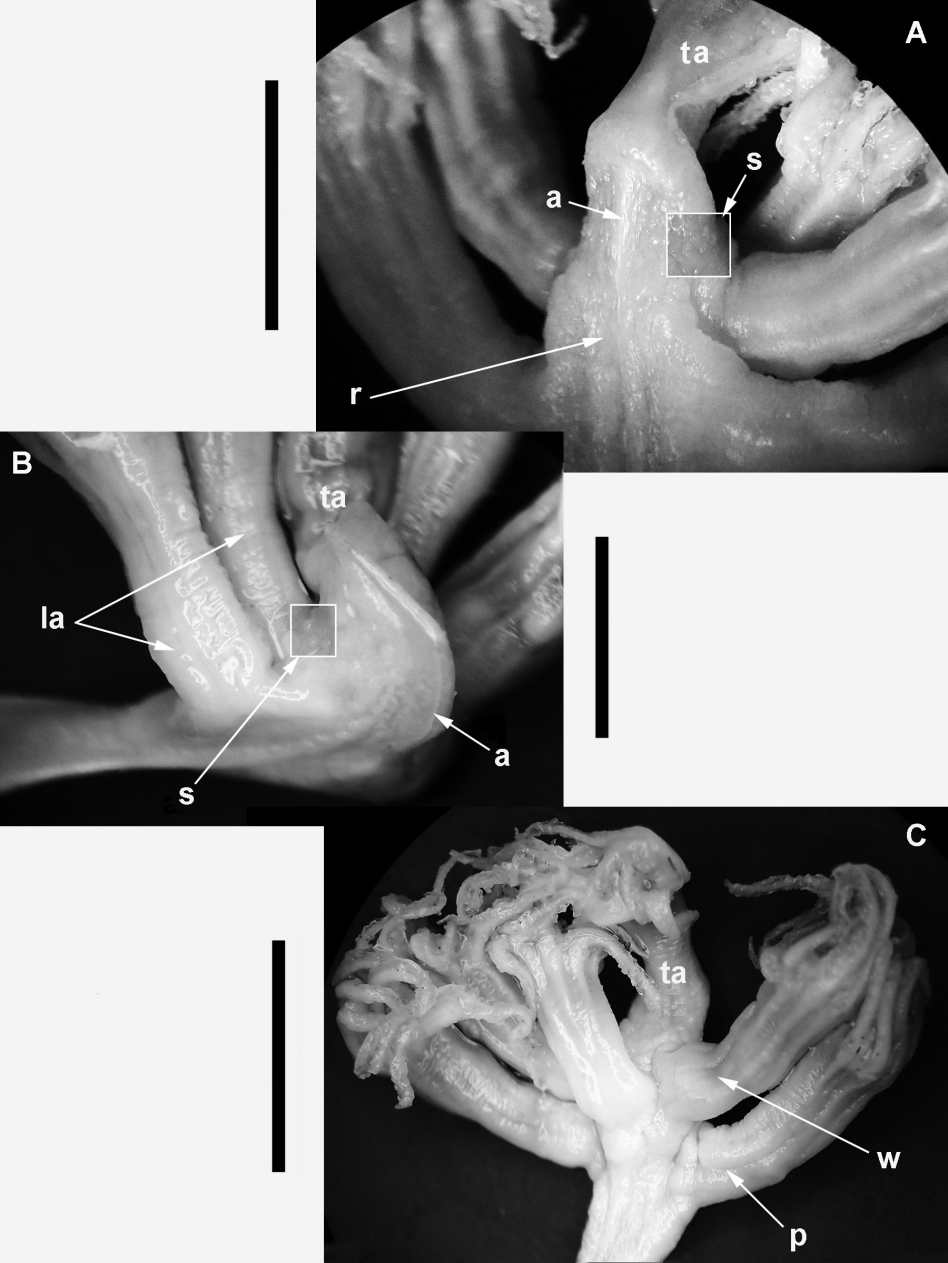
*Porcupinella tasmanica* sp. nov. Micrographs of rachis details **A** view of the distal portion of the rachis (CASIZ 228436), showing placement of a zone of siphonozooids on the lateral side of the rachis between the terminal and lateral autozooids. The axis is visible just below the surface of rachis **B** view of the distal terminus of the rachis (CASIZ 228435), showing the axis visible just below the surface of the rachis. A zone of siphonozooids is apparent on the lateral edge of the rachis, between the terminal and lateral autozooids **C** View of the distal terminal portion of the rachis (CASIZ 228436), showing the terminal autozooid, a whorl of three subtending lateral autozooids, which are in turn subtended by a pair of lateral autozooids. Abbreviations: a – axis; la – lateral autozooid; p – pair of lateral autozooids; r – rachis; s – siphonozooids; ta – terminal autozooid; w – whorl of three lateral autozooids. Scale bar: 4 mm (**A**); 3 mm (**B**); 5 mm (**C**).

Figures 1 , 2 , 3 , 6

###### Species diagnosis.

Mature colony length < 50 mm. Lower rachis region strongly recurved, colonies have an inverted J-shape. Region of strongest curvature forms a laterally flattened keel. Single terminal polyp and generally two pair of lateral polyps congested together in distal region of rachis. Autozooids five to seven in total number. Distal terminus of colony ends in a pointed tip and may form a downward projecting beak-like acute point. Siphonozooids sparsely distributed (< 12 per side), present on upper surface of rachis adjacent and proximal to the lower lateral polyps. Sclerites restricted to minute ovoid bodies in the bulb of the peduncle. Preserved colonies white.

###### Material examined.

***Holotype.*** CASIZ 228434. Western Pacific Ocean, Australia, Tasman Sea, Flinders Marine Park (40.4732 to 40.464S, 149.3967 to 149.4255E); 4114–4139 m; 20 May 2017; coll. RV *Investigator*, IN2017_V03_015_011, Beam Trawl; one whole colony wet-preserved in 95% ethanol. ***Paratype.*** CASIZ 228435, same data as holotype; one whole colony wet-preserved in 95% ethanol.

###### Additional material.

CASIZ 228436, same data as holotype, three whole colonies wet-preserved in 95% ethanol.

###### Habitat and distribution.

Known only from the type locality, Flinders Marine Park in the Tasman Sea, 4114–4139 m in depth (Fig. 6A, B).

###### Etymology.

The specific epithet is derived from “Tasmania” and the Latin suffix –*ica* (belonging to), in reference to the type locality.

###### Description of the holotype.

(Figs 1, 2A). ***Colonial morphology*.** The holotype is 30 mm in length from proximal tip of the peduncle to the distal margin of the keel. If the length of the recurved distal portion of the rachis is included, the total length of entire colony is approximately 46 mm. The keel represents that portion of the colony between the polyp-bearing portion of the rachis and the distal most portion of the peduncle. The keel is somewhat flattened laterally. The maximum height of the keel is approximately 2 mm in the region of strongest curvature. The thin internal axis is clearly distinguishable just below the surface of the upper-most portion of the keel curvature. The distal terminus of the rachis comes to an acute tip and is beak-shaped. The rachis is widest in the middle and tapers at both ends. The peduncle occupies approximately 20 mm of the colony below the proximal portion of the keel. The narrowest part of the peduncle is 0.5 mm in diameter and the widest part is the proximal bulb, approximately 1.5 mm in diameter.

***Polyps*** (Figs 1–3). The lower pair of lateral polyps (autozooids) emanate from opposite sides of the widest part of the rachis, while the bases of the upper pair of lateral polyps are closer together on the narrower distal portion of the rachis. The single terminal polyp emanates from 1–2 mm proximal to the acute tip of the beak near the distal terminus of the colony. The siphonozooids appear as minute, low, hemispherical mounds (<0.2 mm in diameter), and are sparsely distributed on all sides of the rachis from the upper-most portion of the keel to just below the insertion of the terminal polyp. A few siphonozooids are also distributed along the lateral surfaces of the strongly curved portion of the keel, adjacent to the outline of the subcutaneous axis.

***Sclerites*** (Fig. 2 inset). Sclerites are apparently absent throughout the colonies except in tissues of the peduncle, in which there are minute, variably shaped, mostly ovoid bodies, 0.01–0.02 mm in length. These sclerites are difficult to isolate and observe. Sclerites were not observed in other parts of the colony. This is similar to what has been recorded in the type species of the genus, *Porcupinella profunda* (López-González & Williams, 2011: 313.).

***Color*** (Figs 2, 3). The color of colonies in life is unknown, while the color of wet-preserved colonies is white throughout.

***Variation*.** (Figs 2, 3). The five colonies that are known to exist are all from the same collecting station and collecting event in the Tasman Sea. They have an a more-or-less inverted fish hook shape or inverted J-shape and range in length from 27–38 mm (Figs 1, 2). The strongly curved portion of the upper stalk is laterally compressed and flattened, forming a conspicuous keel, with a narrow portion of the axis running throughout the upper-most portion of the curved keel (Figs 1, 2). The axis is present throughout the length of the colonies, and is discernably wider in the peduncle than in the rachis or polyp-bearing upper portion of the stalk, where it is relatively narrow. The proximal end of the short peduncle is somewhat [slightly] swollen and forms an elongated bulb (Figs 1, 2). The total number of autozooids in any given colony is generally five to seven – one terminal polyp and four to six lateral polyps. The siphonozooids are not conspicuous and are relatively sparsely distributed on parts of the rachis and distal portion of the keel (Figs 1, 3A, B). The distal portion of the stalk that contain the polyps is spearhead-shaped or spade-shaped, and ends with an acutely tipped, generally curved beak (Figs 1, 2C). A single terminal polyp emanates from the base of the beak. Two pairs of lateral polyps are oppositely disposed on the spearhead-shaped upper portion of the stalk. (Figs 1–3). Conspicuous sclerites are absent in all parts of the colony, with the exception of minute oval bodies in the peduncle interior. Color of the preserved colonies is white throughout (Figs 2, 3).

###### Differential diagnosis.

**Figure 4.**
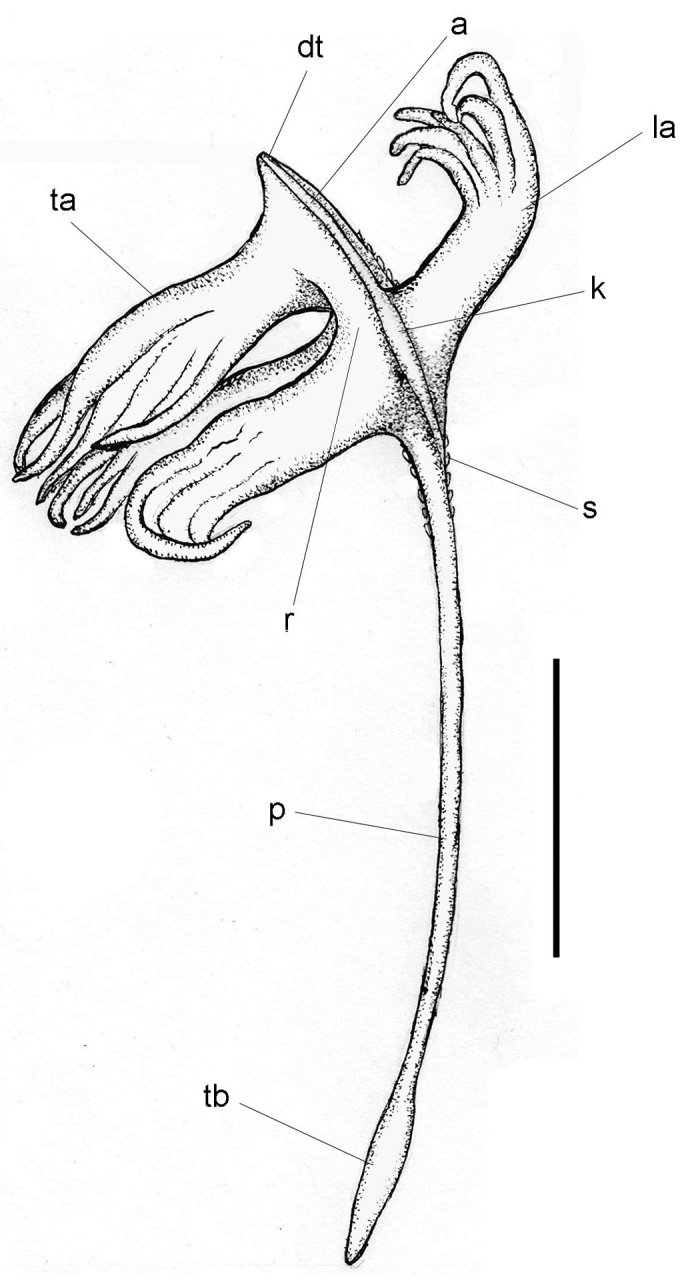
*Porcupinella profunda*. Diagram of external morphology (CASIZ 180424). Abbreviations: a – axis; dt – distal terminus of the colony; k – keel of the stalk; la – lateral autozooid; p – peduncle, r – rachis; s – siphonozooid ta – terminal autozooid; tb – terminal bulb of the peduncle. Scale bar: 10 mm.

**Figure 5.**
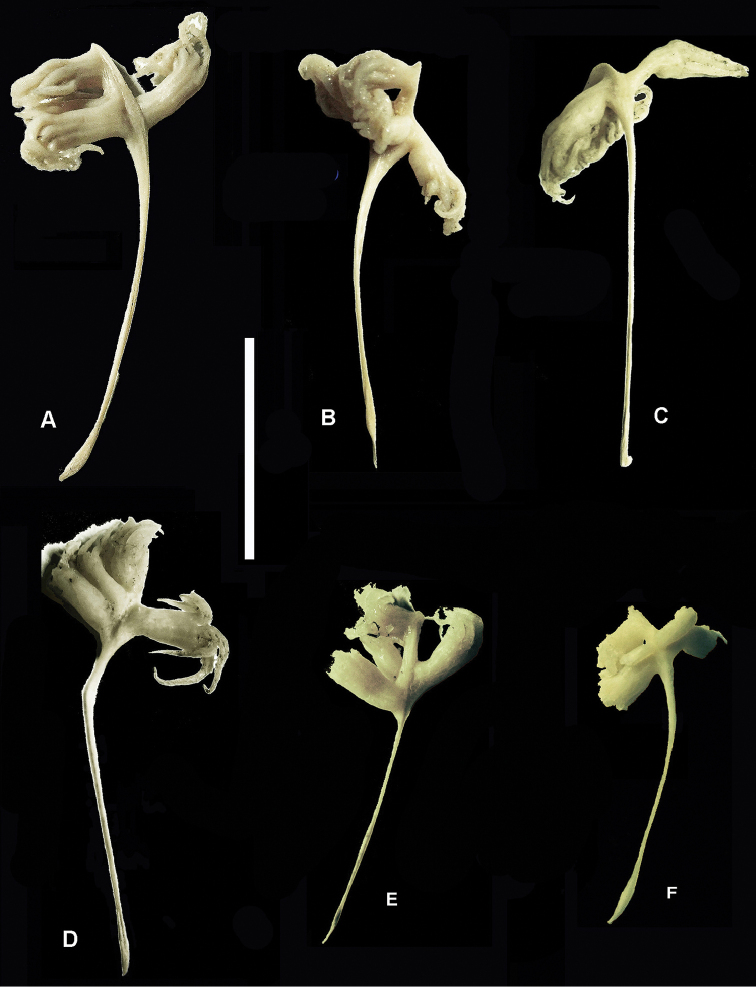
*Porcupinella profunda*. Non-type material **A–B** CASIZ 180424 **C–D** CASIZ 180425 **E–F** CASIZ 180426; Scale bar: 20mm.

*Porcupinella tasmanica* has a strongly recurved growth form, and the autozooids are on the enlarged rachis, which is distal to the keel and separated from it (Figs 1, 2). This is in contrast to the only other known species, *Porcupinella profunda*, which is slightly crescent-shaped to virtually straight, the keel and the rachis are not separated, and the autozooids are contained on the sides of the keel/rachis region (Figs 4, 5).

##### Porcupinella profunda

Taxon classificationAnimaliaPennatulaceaChunellidae

López-González & Williams, 2011

0DEE81B7-C4B0-508A-962D-CB1FE0377564

Figures 4 , 5 , 6

Porcupinella profunda López-González & Williams, 2011: 311; Cordeiro et al. 2020b.

###### Material examined.

(Non-type/Voucher). CASIZ 180424, Northeast Atlantic Ocean, Porcupine Abyssal Plain (48°49.64'N, 16°30.12'W); 4841 m; 17 March 1998; coll. BENGAL cruises (EU Marine Science and Technology Programme, 1994–1998, Bottom Trawl); two whole colonies wet-preserved in 75% ethanol, original fixative 3% buffered formalin. CASIZ 180425, Northeast Atlantic Ocean, Porcupine Abyssal Plain (48°48.30'N, 16°25.97'W); 4839 m; 19 March 1998; coll. BENGAL cruises (EU Marine Science and Technology Programme, 1994–1998, Bottom Trawl); two whole colonies wet-preserved in 75% ethanol, original fixative 3% buffered formalin. CASIZ 180426, Northeast Atlantic Ocean, Porcupine Abyssal Plain (48°47.82'N, 16°40.37'W); 4847 m; 10 January 1998; coll. BENGAL cruises (EU Marine Science and Technology Programme, 1994–1998; Bottom Trawl); two whole colonies wet-preserved in 75% ethanol, original fixative 3% buffered formalin.

###### Habitat and distribution.

**Figure 6.**
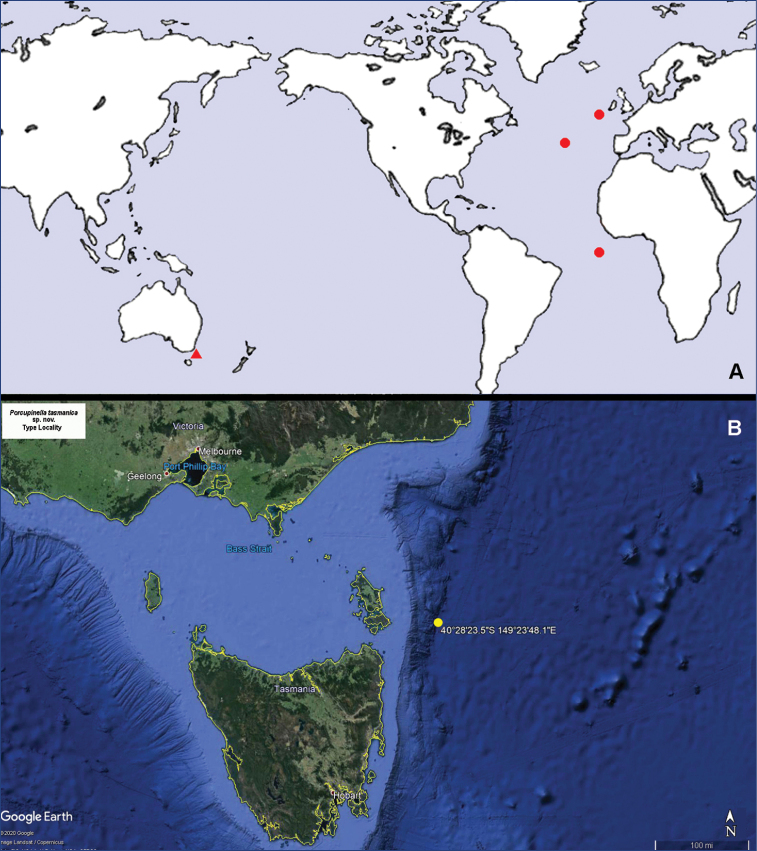
*Porcupinella* spp. **A** world map of distribution. *Porcupinella profunda* (red circle), *Porcupinella tasmanica* sp. nov. (red triangle) **B** detailed map of the type locality of *Porcupinella tasmanica* sp. nov. (yellow circle = type locality). Map data 2020 Google.

Recorded from the eastern Atlantic Ocean from the Porcupine Abyssal Plain (southwest of Ireland), south to equatorial latitudes, 4510–5300 m in depth. (López-González and Williams 2011: 314) (Fig. 6A).

###### Description.

The six whole colonies from the Porcupine Abyssal Plain that were examined for this study range in length from 29–41 mm (Figs 4, 5). The colonies are slightly crescent-shaped to more-or-less straight. The polyp-bearing portion of the upper stalk is somewhat widened, forming a keel, where the relatively large lateral polyps are arranged (Fig. 4). The axis is present throughout the length of the colonies, and is discernably wider in the rachis and is very close to the surface, which provides more rigidity to the polyp-bearing portion of the rachis. The total number of autozooids in any given colony is generally five – one terminal polyp and four lateral polyps. The siphonozooids are not particularly numerous and are presented as low rounded mounds on the surface of the rachis between the lateral and terminal autozoods and just proximal to the lateral autozooids. The distal portion of the stalk that contain the polyps is narrowly spearhead-shaped, and ends with a pointed tip or beak (Figs 4, 5A, B). A single terminal polyp emanates from the base of the distal terminus. Two pairs of lateral polyps are oppositely disposed on the spearhead-shaped upper portion of the stalk. (Fig. 5). The proximal end of the short peduncle is somewhat [slightly] swollen and forms a relatively narrow, elongated bulb (Figs 4, 5A, F). Conspicuous sclerites are absent in all parts of the colony, with the exception of extremely small ovals in peduncle interior. The color of wet-preserved colonies is uniformly white throughout (Fig. 5).

###### Remarks.

At least twenty-five collected colonies are known, thirteen of which are deposited at the Muséum national d’Histoire Naturelle (MNHN), six at the Biodiversidad y Ecología de Invertebrados Marinos of the University of Seville (BEIM), and six at the California Academy of Sciences (CAS).

## Discussion

The pennatulacean family Chunellidae was described by Kükenthal (1902) to include two monospecific genera of deep-water sea pens found off the coast of Somalia, in the western Indian Ocean – *Chunella* and *Amphiacme*. Kükenthal (1902) originally named the latter genus *Amphianthus* Kükenthal, 1902, which was preoccupied by a genus of anemone and therefore changed the name to *Amphiacme* (Kükenthal, 1903). The geographic ranges of both species, *Chunella gracillima* and *Amphiacme abyssorum* were extended down the coast of east Africa to South Africa (Williams 1990, 1992). Subsequently, the genus *Chunella* was recorded from Indonesia (Hickson 1916). Therefore, *Chunella* has Indo-West Pacific in distribution, while *Amphiacme* is apparently limited to the east coast of Africa.

To date, a robust and reliable molecular analysis has not been accomplished to treat the phylogenetic placement of the genus *Porcupinella*, due in part to the difficulty of obtaining suitable material for examination at present. One effort has been attempted, however uncertainty exists regarding the valid identification of material used. It was tentatively identified as a species of *Porcupinella*, but may actually represent the genus *Umbellula* Gray, 1870 (U. Ganguly, pers. comm.). Since molecular data for taxa within the family Chunellidae does not exist at present, the elucidation of phylogenetic affinities of the genus *Porcupinella* would require acquiring molecular data for the type species of the genus *Chunella* (C. McFadden, pers. comm.), which due to the rarity of collected material and the difficulty of obtaining fresh material, is not feasible at present. However, such an overall reliable analysis would potentially corroborate or contradict the placement of the genus in the family Chunellidae and could also elucidate its phylogenetic position among other deep-sea pennatucaeans.

The genus *Porcupinella* is here included in the family Chunellidae based on several morphological characters, such as possessing a single terminal polyp at the distal end of the rachis, which varies within the family from functional and fully formed (*Porcupinella*), to modified and zygomorphic (*Amphiacme*), to highly reduced and rudimentary (*Chunella*).

Morphological similarities between some species of *Umbellula* (family Umbellulidae) and chunellids such as *Porcupinella* have previously been discussed by López-González and Williams (2011).

### Key to the world species of the pennatulacean family Chunellidae

**Table:** 

1	Terminal polyp absent	*Calibelemnon* spp.^*^
–	Terminal polyp present	**2**
2	Pairs or whorls of lateral polyps separated by conspicuous areas of bare rachis	**3**
–	Pairs of lateral polyps congested in distal region of rachis near the terminal polyp	**4**
3	Terminal polyp rudimentary and highly reduced, tentacles absent	**5**
–	Terminal polyp fully developed, zygomorphic and laterally compressed, tentacles present	***Amphiacme abyssorum***
4	Colonies crescent-shaped to more-or-less straight. Polyps emanate from the keel-bearing portion of rachis	***Porcupinella profunda***
–	Colonies inverted J-shaped, markedly recurved in distal portion of rachis. Polyps remote from the bare keel	***Porcupinella tasmanica***
5	Polyps three per whorl	***Chunella gracillima***
–	Polyps two or four per whorl	**6**
6	Polyps in pairs (two per whorl)	***Chunella biflora***
–	Polyps four per whorl	***Chunella quadriflora***

## Conclusion

Of the thirty-seven extant genera of pennatulaceans the three deepest known are *Kophobelemnon* Asbjørnsen, 1856 (36–4900 m), *Porcupinella* (4114–5300 m) and *Umbellula* (210–6260 m) (Williams 1995, 2019).

Three species of *Chunella* have been described and differentiated on the basis of number of polyps per whorl, which may be due to intraspecific variation, and thus the taxonomic limitations of the genus is dubious at present (Cordeiro et al. 2020b). *Chunella gracillima* Kükenthal, 1902, is the type species of the genus (Kükenthal 1915: 45).

Two species of *Porcupinella* are known, the type species, *P. profunda*, from the Atlantic Ocean, and *P. tasmanica* sp. nov. from the southwestern Pacific.

## Supplementary Material

XML Treatment for Porcupinella tasmanica

XML Treatment for Porcupinella profunda

## Footnotes

Similarities between members of the genera Calibelemnon Nutting, 1908, Umbellula and other chunellids have been treated or at least mentioned in some way by several authors including Hickson (1916), Williams (1995) and Kushida and Reimer (2020). Calibelemnon has been included in this key since they do have bare areas of rachis between the polyp pairs as in Chunella and Amphiacme, and do not have conspicuous sclerites as in all chunellids. They do not possess a terminal polyp, and so are not considered as part of the Chunellidae, but are recognized as part of the family Scleroptilidae.
